# The emerging roles of the gut microbiome in allogeneic hematopoietic stem cell transplantation

**DOI:** 10.1080/19490976.2021.1966262

**Published:** 2021-08-30

**Authors:** Lam T. Khuat, Maneesh Dave, William J. Murphy

**Affiliations:** a Department of Dermatology, School of Medicine, University of California, Davis, CA, USA; b Division of Gastroenterology, Department of Internal Medicine, School of Medicine, University of California, Davis, CA, USA; c Department of Internal Medicine, School of Medicine, University of California, Davis, CA, USAs

## Abstract

Allogeneic hematopoietic stem cell transplantation (allo-HSCT) is used for the treatment of hematologic cancers and disorders. However, graft-versus-host disease (GVHD) in which the donor immune cells attack the genetically-disparate recipient is a significant cause of morbidity. Acute GVHD is an inflammatory condition and the gastrointestinal system is a major organ affected but is also tied to beneficial graft-versus-tumor (GVT) effects. There is increasing interest on the role of the microbiome on immune function as well as on cancer progression and immunotherapy outcomes. However, there are still significant unanswered questions on the role the microbiome plays in GVHD progression or how to exploit the microbiome in GVHD prevention or treatment. In this review, concepts of HSCT with the focus on GVHD pathogenesis as well as issues in preclinical models used to study GVHD will be discussed with an emphasis on the impact of the microbiome. Factors affecting the microbiome and GVHD outcome such as obesity are also examined. The bridging of preclinical models and clinical outcomes in relation to the role of the microbiome will also be discussed along with possibilities for therapeutic exploitation.

## Current concepts in hematopoietic stem cell transplantation

Hematopoietic stem cell transplantation (HSCT) is primarily used for the treatment of hematological malignancies such as leukemia and lymphoma, but also for hematopoietic disorders^1^. Before receiving the donor cells, recipients are treated with cytoreductive or immunosuppressive conditioning regimens including either total body irradiation, chemotherapy, or a combination of both to eradicate the malignant or abnormal cells, create a niche for the donor hematopoietic stem cells (HSCs) as well as facilitate engraftment and reconstitution.^2^ There are two principal types of HSCT: allogeneic HSCT (allo-HSCT), in which there is genetic disparity between the donor HSCs and the recipient, and autologous HSCT (auto-HSCT) in which the HSC’s are from the recipient. The primary advantage of allo-HSCT is the potent antitumor response due to a graft-versus-tumor (GVT) effect generated by donor T cells to the tumor. However, this beneficial GVT is also tied to one of the primary disadvantages of an allo-HSCT revolving around the development of recipient tissue damage in the gastrointestinal (GI) tract, skin, and liver due to the attack by the allo-reactive donor T cells, called graft-versus-host disease (GVHD). GVHD represents a major cause of morbidity following allo-HSCT. The extent of genetic disparity, the types and extent of cytoreductive conditioning used on the recipient as well as the presence of co-morbidities all impact the occurrence and severity of GVHD.^3^ GVHD represents a major limitation in allo-HSCT and necessitates the need for immunosuppression which also impacts the beneficial GVT effects resulting in higher relapse.

The cytoreductive conditioning regimens play a crucial role in HSCT outcome but also result in significant toxicities, particularly in older patients. The myeloablative regimen consists of intensive radiation therapy alone or in combination with chemotherapy but can result in significant GI, hepatic and pulmonary toxicities also fueling the GVHD processes. Therefore, non-myeloablative immunosuppressive conditioning using agents such as fludarabine as well as lower doses of cytoreductive agents are now used in order to reduce toxicities and facilitate engraftment of donor HSCs, especially for children or older patients and this has greatly increased application of allo-HSCT to these patient cohorts.^4,5^ Outside of direct tissue attack, delayed hematopoiesis and immune reconstitution post-transplant which also result during GVHD are additional life-threatening complications of allo-HSCT due to susceptibility to opportunistic infections. Immune cell reconstitution is critical but is also dependent on the age of the recipient but NK cells (30–100 d) followed by T cells (from 100 d but can take years, especially in older patients), and B cells (1–2 y) all impact susceptibility to opportunistic infections as well as relapse.^6^ Increased susceptibility to bacterial infections such as *Pseudomonas, Enterobacter, Klebsiella*, parasites, fungi, and reactivation of latent viruses such as Cytomegalovirus, Herpes Simplex, and Epstein Barr virus all rise.^7^ To counteract these conditions, the uses of hematopoietic growth factors (granulocyte colony-stimulating factor, G-CSF) to promote myeloid recovery, administration of prophylactic antibiotics, and anti-viral therapies (for CMV reactivation) are routinely applied.^8,9^ Patient demographics are being increasingly appreciated as factors such as obesity, diet, age and other co-morbidities clearly affect the allo-HSCT outcome but are still poorly understood as well as the impact of these different prophylactic regimens on the various aspects of HSCT and recovery.

## Graft-versus-host disease

Although allo-HSCT is a potential treatment for hematological malignancies, GVHD still remains one of the most difficult obstacles for the success of this approach.^3^ GVHD is the immunological phenomena mediated by the donor-derived T cells in the graft targeting the major histocompatibility complex (MHC) and minor-MHC antigen differences (which in allo-HSCT exist despite more sophisticated typing and matching to ensure greater compatibility) and immunosuppressed recipient resulting in host multi-organ organ attack including the GI system, lung, liver, skin resulting in tissue damage, organ failure, and mortality. GVHD is the cause of 15–30% of deaths post-allogeneic HSCT.^10^ GVHD prevention can be successful by simply removing donor T cells from the HSC graft but unfortunately, it also abrogates beneficial GVT and the dominant procedures use T cell-replete grafts. An effective means to treat ongoing GVHD outside of blanket immunosuppression is still lacking especially with severe GVHD due to the nature of a “cytokine storm” that occurs and these immunosuppressive regimens also impact GVT and therefore relapse. Therefore, understanding the mechanisms of GVHD induction as well as evaluation of multiple approaches to ameliorate this disease is in need.

There are two principal types of GVHD with distinct features in pathobiology and clinical phenotypes: acute and chronic GVHD. Clinically, acute GVHD (aGVHD) usually appears at the first 100 d after transplant, while chronic GVHD (cGVHD) was classically defined as occurring later although this is now shown not to be absolute and both processes can also occur at the same time.^11,12^ The pathologic processes of aGVHD and cGVHD are clearly distinct. aGVHD is primarily an inflammatory disease and is due to many factors such as tissue toxicities arising after conditioning regimen which culminates with donor T cell-mediated target tissue damage due to MHC disparities and massive pro-inflammatory cytokine release originally called a “cytokine storm” and which is extremely difficulty to treat.^13^ The primary aGVHD target organs are the GI tract or gut, liver, lung, and skin with severe acute gut GVHD representing a major cause of early morbidity. It is the pro-inflammatory cytokine storm consisting of IL-1, TNF, IL-6, and other cytokines and recruitment of innate cells such as macrophages which amplify the process that makes aGVHD extremely difficult to control or treat. In contrast, cGVHD typically results later after HSCT and is associated with tissue fibrosis resembling an autoimmune-like syndrome attacking skin and mucosal tissues.^3^ As opposed to aGVHD, cGVHD is associated with a dominant role for donor B cells in its progression. The last phase of cGVHD involves in tissue fibrosis regulated mainly by macrophages with transforming growth factor β and platelet-derived growth factor α. These pathological changes can severely impact quality of life and cGVHD is notoriously difficult to treat as it can become resistant to steroids or immunosuppressive regimens resulting in extremely limited options for treatment. It is important to note that these processes are not exclusionary and a patient can present with both types necessitating determination by biopsy.

Cytoreductive conditioning regimens are the predominant cause of mucositis early post-HSCT, followed by the development of acute GVHD.^14^ Radiation and chemotherapeutics such a cyclophosphamide cause extensive DNA damage and apoptosis in the GI tract, along with reduced proliferation of intestinal stem cells. This leads to an increase in intestinal permeability, which results in bacterial translocation from the microbiome in the gut, culminating in systemic infection and reduced survival post-HSCT.^13^ Endotoxin from bacterial translocation further active myeloid cells via toll receptor engagement and fuel the allo-reactive processes of the donor T cells and exacerbate the gut damage causing the cascade to progress. The gut tissue damage by the conditioning regimens, also promote recruitment of allo-reactive donor T cells to the intestinal tract.^15^ Acute GVHD with the destruction of the intestinal mucosa leads to a failure of fluid absorption, particularly in the ileum, and voluminous diarrhea in patients.^14^ One of the earliest symptoms of GI GVHD is the tissue damage happened in duodenum, leading to the early bleeding, appetite loss, nausea, vomiting, and weight loss.^16–18^ The continuous development and use of anti-fungi, anti-viral, and GVHD prophylaxis has helped in reducing the incidence of severe GI bleeding after HSCT.^19^ The pathology of aGVHD in the gut is distinctive with neutropenic enterocolitis diagnosed by the thickening of the ileum and colon wall using abdominal computed tomographic (CT) scan.^20^ CT scan with intravenous and oral contrast confirms thickened bowel segments in patients with acute GVHD.^21^ Similar to CT scan, magnetic resonance enterography (MRE) also could be used as an alternative method for assessing GI acute GVHD with its capability to detect long-segment bowel wall thickening, submucosal edema, and mucosal hyperemia.^22^ Treatment options primarily consist of blanket immunosuppression although more targeted approaches including cytokine blockade or blocking T cell homing to the gut via blockade of specific integrins are being evaluated.

## Graft-versus-tumor effect

Allo-HSCT is used as a cancer immunotherapy because it generates the GVT effect in which donor-derived cells attack the host hematologic malignant cells.^1,23,24^ Although GVHD and GVT effect share several biology processes such as antigen-presenting cell activation and donor T cell activation possibly to similar if not identical antigens, it has been difficult even in preclinical models to delineate the two processes which nonetheless remains the primary goal in studies. Lympho-depletion has been shown to enhance the efficacy of adoptively transferred tumor-specific CD8 + T cells by using antibody to remove the γ_C_ cytokine-responsive endogenous cells.^25^ Interestingly, in cancer immunotherapy, depletion of the patient’s lymphocytes has been increasingly studied to improve efficacy of adoptive immune cell transfer. Lympho-depleting conditioning regimen prior to adoptive cell transfer in patients with metastatic melanoma significantly improved the efficacy of therapy with in vitro expanded tumor-infiltrating lymphocytes.^26^ These cytoreductive conditioning regimens are also routinely applied with chimeric antigen receptor (CAR)-T cell therapy and similar toxicities are being observed including impact on cytokine storm or “cytokine release syndrome.”^27,28^ Outside of merely creating a niche for the transferred T cells, with T cell immunotherapy outside of HSCT, it has been postulated that bacterial translocation due to conditioning regimen potentially contributed to improve efficacy by activating dendritic cells augmenting the function of adoptively transferred CD8 T cells.^29^ It has been demonstrated that an intact microbiome is essential to maintain anti-tumor effects in preclinical models. Use of gnotobiotic or germ-free mice as well mice treated with antibiotics all have reduced responses to immunotherapies indicating an important role of the microbiome in immune regulation and function.^30,31^ Additionally, it was observed that certain types of bacteria potentially contribute to modulate the clinical outcomes with immunotherapies. *Bifidobacterium* spp. is associated with delayed tumor progression and increased responses to programmed death-ligand 1 (PD-L1) checkpoint blockade.^32^ Using another preclinical model, *Bacteroides* spp. is found to be required for optimal anti-tumor responses of cytotoxic T-lymphocyte-associated protein 4 (CTLA-4) checkpoint blockade.^33^ These results indicate that the microbiome can play pivotal roles in cancer immunotherapy efficacy regarding T cell immunotherapies as well as potential GVT effects post-allo-HSCT.

## Significant differences between HSCT in murine models and humans

The inbred laboratory mouse model is the most common preclinical model used to study allo-HSCT. The development of inbred laboratory mouse husbandry and genetic modification tools facilitated the broad use of murine models of GVHD and GVT. Mice are inbred (genetically identical), fed with controlled diets, and housed in highly regulated specific pathogen free (SPF) environments which provide researchers the ability to generate more reproducible data. However, these factors also give rise to significant discrepancies between the clinical scenario which is not as controlled given that humans are outbred and exposed to multiple pathogens throughout life and often as they age, have the presence of co-morbidities including obesity. Another one of these variables that is increasingly appreciated concerns the microbiome of laboratory mice and its difference between humans. Outside of clear species differences and dietary habits, there are multiple studies providing evidences of how mouse gut microbiota shifts in various housing conditions and affects the immune system. In addition, compared to mice housed under SPF conditions, the use of germ-free mice often used in microbiome transfer studies, lack all microorganisms and have various immunological abnormalities with significant defects in immune development. These include deficits in development of lymphoid organs, altered mucosal immunity, impaired innate cell number and function, and adaptive responses being in a naïve state as well as demonstrated decreases of total CD4+ helper T cells while shifting to T_H_2 phenotype.^34,35^ Gnotobiotic mice are animals with defined microorganisms and are also used in studies to study the impact of particular bacterial species. However, the complexity of handling protocols, quality control of bacterial colonization, and extremely high cost are the main limitations for extensive application of this model in current research and similar immune deficits.^36–38^ It is also important to keep in mind that, even under SPF housing conditions, mice obtained from different vendors display distinct microbiome profiles which has been demonstrated to significantly affect their responses to cancer immunotherapies.^32^ Importantly, these differences were found to be normalized when mice are co-housed or even bedding from one are transferred given that mice exhibit coprophagy (the eating of their feces) which normalizes the microbiome within a cage.^32^ Interestingly, feral mice and mice obtained from pet stores have extensive bacterial and pathogen exposure and present with notably more diverse microbiota than SPF mice. Furthermore, the immunological profile of such mice has been demonstrated to more appropriately model the complexity of human immune system demonstrating the importance of the microbiome on immune functions but also the complexities in attempting to model reflecting the human condition.^39^

**Figure 1.**
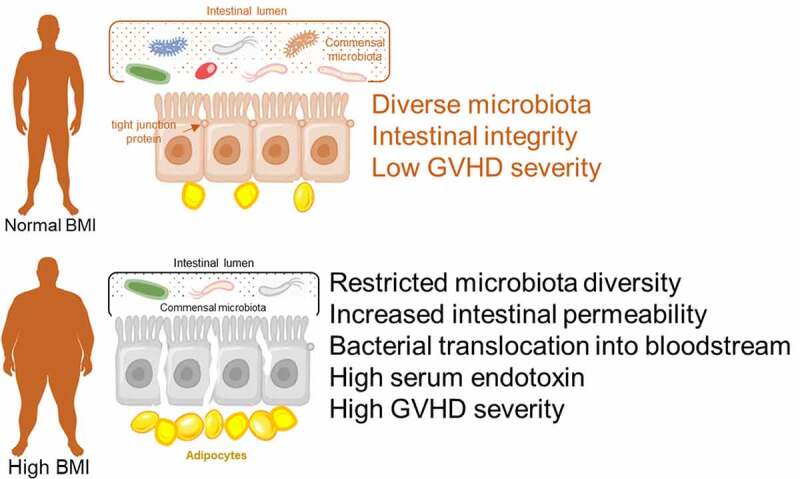

Outside of the not insignificant species differences, human populations exhibit considerable MHC diversity, wide ranges in age and different pathogen exposure as well as the existence of preexisting conditions (i.e. diabetes, obesity) all of which can be influenced by the microbiome and have profound immunological consequences. Both obesity^40^ and aging^41^ are associated with a heightened inflammatory state which predisposes individuals to after HSCT to cytokine-induced pathologies due to increased gut permeability and bacterial translocation^42,43^ and condition regimen complications targeting the GI tract (i.e. radiation toxicity and bacterial translocation).^44^ Obesity (BMI > 30) has reached pandemic proportions in the U.S. with greater than one-third of U.S. adults are obese according to Center for Disease Control and Prevention. Obesity is associated with meta-inflammation exacerbated by metabolic complications such as glucose intolerances, diabetes, hypertension etc. The inflammation in the obese environment is also fueled by adipocyte factors and the effects on the immune system have been called “inflammaging” due to suppressed adaptive immune responses.^45^ This is in part due to alteration of immune subsets^46,47^ as well as increased production of inflammatory cytokines such as IL-1β, TNF-α, IL-6 from macrophages, monocytes, and T cells.^48^ Obesity also impacts the microbiome with less diversity being reported.^49,50^ Obesity of the recipient has been shown to correlate with poor outcomes after allogeneic HSCT in both mouse preclinical models and human clinical outcome data^50^ in which high BMI was associated with a significantly greater risk of grade II–IV acute GVHD^51^ (Figure 1). However, there are also clinical studies suggesting that obesity was associated with a higher survival rate after allo-HSCT^52^ and auto-HSCT.^53^ As there can be significant differences in HSCT procedures (such as patient selection, conditioning, regimens, stem cell sources, immunosuppression applied) as well as the presence of co-morbidities such as cancer, all of these can impact outcomes.^54^ Apart from obesity, aging also plays a critical role in inducing microbiota changes. The decreased abundances of *Bifidobacterium* and *Lactobacillus*, together with an increased abundance of *Enterobacteriaceae* are the key microbiota shifts in elderly individuals.^55^ Possible reasons for microbiome shifts in aging could be explained based on diet adjustment, lessened exercise, reduced mobility, residence locations, less muscle mass (sarcopenia), etc.^56^ Although obesity and aging correlate with restricted microbiota diversity in mouse and human^50,55,57,58^ and the less-diverse microbiome profile has been proved to be associated with poor outcomes after HSCT,^59–61^ recent studies suggested that there are certain types of bacteria that could serve as beneficial factor for health and longevity.^56,62^ *Bifidobacteria* supplementation reduced the accumulation of aging biomarkers (carbonyls and lipofuscin),^63^ while transfer of *Christensenella minuta* to germ-free mice significantly reduced adiposity gain.^64^ Thus, there is an interplay between the gut microbiome on body weight gain and immune outcomes but also in reverse where the diet can impact the microbiome.

Humans also have been exposed to various immunological challenges and pathogens throughout life (i.e. cytomegalovirus, EBV) that significantly affect HSCT outcomes. Clinical data have suggested that obese patients are at higher risk of infection-associated complications and transplantation-related toxicity post-HSCT, especially in allogeneic HSCT.^65,66^ These data indicate the necessity to modify our current murine HSCT models to be more reflective of human conditions (Table 1). One excellent example of this is the study with co-housed laboratory mice with pet store mice gave rise to significant alterations in the immune profile of laboratory mice with increased resistance to infection and altered T cell kinetics.^39^ The problem arises with regard to the costs of using so-called “dirty” mice and lack of ability to therefore control all the variables allowing for reproducible data, particularly between different laboratories.

**Table 1.** Comparison of HSCT in mice and humans

	Mice	Humans
Genotype	Inbred (genetically homogeneous)	Outbred with tremendous human leukocyte antigen (HLA) diversity
Age	Predominantly young (8–12 weeks old), equivalent to early adolescence in humans	Variable age
Environment	specific-pathogen-free (SPF)	Numerous pathogen exposures (acute, chronic and latent)
Microbiome	Husbandry-related factors such as mouse transfer, water decontamination could affect microbiome change^67,68^	Preexisting disease (i.e., obesity^50,57,58^), environmental exposure, and antibiotic use all have effects on the human microbiome^69^
Diet	Changes in diet can alter the intestinal microbiome^70–72^	Diet types (omnivorous, vegan, and vegetarian) determine microbiome profiles^73^
Recipient health status	Healthy lean (body weight below 30 g)	Highly variable, with an increasing obesity (BMI >30 kg/m^2^) and preexisting diseases
HSCT conditioning regimens	Lethal dose of total body radiation (single or split doses), chemotherapy rarely applied	Myeloablative and non-myeloablative regimens
HSCT application	Modeling of HSCT for human cancers, including xenogeneic transplant models	Treatment of a variety of disease states ranging from cancer to hematopoietic disorders
GVHD pathogenesis	Donor bone marrow cells do not give rise to GVHD. Donor T cells (mostly naïve T cells) are required to induce GVHD.	Adult HSC sources are sufficient to cause GVHD depending on the level of HLA compatibility and the conditioning regimen used
GVHD type	Mostly either acute or chronic, crucially dependent on strain combination used and the type of conditioning.	GVHD can be mixed (acute and chronic)
Graft-versus-tumor (GVT) effect	Numerous studies of approaches to enhance GVT effect and limit GVHD, although the emphasis is on GVHD prevention or treatment and only short-term results	Emphasis has been to improve GVT either through adoptive cell therapies or improved immune reconstitution following HSCT, without exacerbating GVHD

In order to mirror the clinical HSCT scenario more accurately, large-animal models, primarily in canines and non-human primates, have been studied and represent the key models for GVHD studies and the development of treatment protocols for HSCT. However, extended use of these models are hampered due to high costs, difficulties in experimental control of MHC disparities, limited validated immune reagent availability and immune monitoring capabilities, time and limited sample size, as well as difficulty to use in cancer studies which are the predominant reasons for allo-HSCT. GI tract GVHD in rhesus macaques has been characterized with diarrhea as a clinical symptom, significant lymphocyte infiltration and loss of normal tissue architecture with mucosal damage with histology assessment and represents a useful GVHD model.^74–76^ In canine models, GI tract acute GVHD displayed a distinct punctate GI hemorrhage with blunted villous architecture, sloughing, mucosal destruction progressing from crypt abscess formation to denudation, and CD3 infiltration representing typical GI acute GVHD symptoms also representing a useful model but mechanistic studies are extremely difficult to perform.^77,78^ Thus, both small animal and large animal models offer advantages and disadvantages in HSCT studies (Table 1) and these need to be constantly taken in consideration when evaluating data and attempting to extrapolate to the clinical situation.

## Methodological approach to study intestinal microbiota

Given the predominance of gut GVHD and the importance of the microbiome on immune functions, particularly in the gut, there has been considerable interest in defining the gut microbiome in both preclinical and clinical samples. The most commonly used method in microbiome studies uses sequencing of 16S ribosomal RNA (rRNA), the highly conserved gene in all bacteria. Sequencing of regions of hyper-variability in 16s rRNA gene helps us identify different bacterial taxa but this technique could result into inaccuracies at species level classification. The most common hyper-variable regions in 16S rRNA is V3-V4 region. A simple DNA sequencing procedure involves bacterial DNA extraction from samples, bacterial DNA amplification from variable regions of 16S rRNA using polymerase chain reaction (PCR), library preparation, DNA sequencing, bioinformatics, and biostatistics analyses.^79,80^ Shotgun next-generation metagenomic sequencing (shotgun NGS sequencing) is also a DNA sequencing method but amplifies all genomic DNA in a sample (including bacterial DNA and non-bacterial DNA) by gene fragmentation, tagging, PCR amplification, sample pooling in equal proportions, DNA sequencing, and bioinformatics analysis to classify results into taxonomic levels.^81^ Because of broader sequencing capabilities, shotgun NGS sequencing allows researchers to identify multiple bacteria, fungi, viruses and many other types of microorganisms in their samples.^82^ Diversity is one of the most important parameters in microbiome study. Microbiome diversity has been well-established as a “biomarker” for HSCT outcome. High microbiome diversity correlates with better survival rate, lower incidence and severity of GVHD.^59,60,83,84^ Alpha-diversity (definition of the species composition within samples) and beta-diversity (quantification of the overall compositional differences between groups of subjects) are key factors to study the gut microbial ecosystem.^85^ In alpha-diversity, Shannon diversity index was used to describe evenness and diversity by measuring both the number of species and the inequality between species abundances. In contrast, Shannon evenness index is independent of species richness and provides information about how evenly the microbes are distributed in a sample. Beta diversity represents the differences between microbial communities from different environments with the main focus on the differences in taxonomic abundances from different samples.^86^ Because of new molecular techniques, statistic and bio-informatics analysis methods are discovered every few years; microbiome characterization is expected to reach the next level soon with potential linkage with microbiome alteration factors such as antibiotics, strain dynamics, and homeostasis.

## Microbiota modification and HSCT outcomes

**Figure 2.**
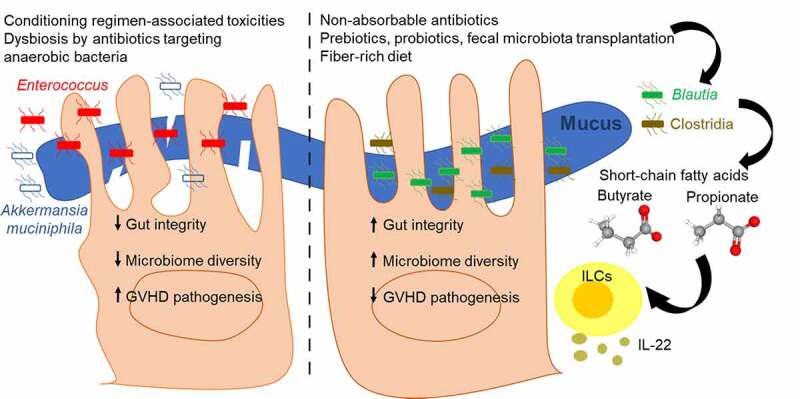

Commensal bacteria are the results of the co-evolution and symbiotic relationship between the host and microorganisms. They impact the host metabolism as well as provide protection against pathogenic bacterial growth. A diverse microbiota is required to maintain host-microbe homeostasis in various environmental changes because species-rich bacterial communities could compensate for missing ones. In HSCT, together with microbiome diversity as an indicator for outcomes,^60^ some pathogenic bacterial taxa have been shown to increase in the GVHD recipients such as Enterococcaceae, *Akkermansia muciniphila*, and Lactobacillales.^50,61,84,87^ In contrast, Blautia has been shown as the beneficial bacterial genus for HSCT^88^ as well as the correlation of increased GVHD with reduced abundances of some bacterial genera such as *Faecalibacterium, Bacteroides, and Parabacteroides*.^89^ Certain bacterial taxa also have been shown to contribute to GI tract recovery after radiation. *Lachnospiraceae* and *Enterococcaceae* are essential to maintain GI tract integrity and facilitate immune reconstitution long-term after radiation exposure.^90^ Therefore, multiple selective microbiota alteration approaches have been extensively studied to ameliorate GVHD and improve HSCT outcomes (Figure 2). It is important to note that the conditioning regimens applied in HSCT often involve cytoreductive conditioning in the form of radiation and chemotherapeutics which cause significant damage to the GI system thus exacerbating the impact of the microbiome on outcome, particularly during the initial period of immune deficiency in the recipient immediately following HSCT.

## Antibiotics

Following the rapid development of cutting-edge techniques for microbiota analysis, the important role of the microbiome in health and diseases has been extensively revealed. These achievements have opened many research directions to modify microbiota component to establish highly effective microbiome therapies^91^ in pre-clinical models and in clinics. Early pre-clinical studies indicated a significant reduction of GVHD with antibiotics^92^ and transplantation in germ-free conditions.^93^ However, there are also conflicting clinical data demonstrating poorer outcome in patients receiving prophylactic antibiotics. We and others demonstrated in murine HSCT models that prior administration of broad spectrum antibiotics reduced acute GVHD post-allogeneic HSCT in part due to significantly decreased bacterial translocation into bloodstream.^50,94^ Interestingly, increased expression of MHC class II in the intestinal epithelial cells after radiation was also reduced, likely affecting donor T cell priming for GVHD induction in HSCT.^95^ Other possible mechanisms include reduced migration of neutrophils^96^ or donor T cells^50^ into the mesenteric lymph nodes where priming for gut GVHD occurs. However, there is also recent evidence that antibiotics with specific activity targeting the ribosome demonstrated a direct immunosuppressive role against T_H_17 cells, suggesting that antibiotics with no specific target on bacteria can directly exert immunological regulatory effects so caution must be exercised before assuming that the microbiome was the mechanism underlying the effects.^97^

The microbiome recovery after antibiotic treatment and HSCT is another parameter which can be variable and is impacted by factors intrinsic to the patient as well as diet and regimens applied all affect the gut and microbiome repopulation dynamics. It was reported that the recovery of the gut microbiota started around day 50, but even by day 100 post-HSCT, the composition and bacterial abundance were less diverse compared to the pre-transplant microbiota.^98^ In allogeneic HSCT patients, sterile food, antibiotic treatment, and skin cleansing significantly reduced mortality.^99^ Promising results representing the correlation of complete gut microbiota decontamination with better outcomes post-HSCT also came from studies using prophylactic antibiotic administration. Vossen et al. used the combination of neomycin, polymyxin B, cephaloridin, and amphotericin B to target all bacteria, yeasts, and fungi and demonstrated reductions in both infections and GVHD in children.^100^ A follow-up study comparing the efficacy of successful and unsuccessful microbiota decontamination in children before and after HSCT in GVHD prevention also suggested the remarkable reductions of moderate and severe acute GVHD in patients with complete microbiota decontamination.^101^ It is important to notice that although the use of broad-spectrum antibiotics dramatically protected patients against infection and transplant-related mortality, antibiotics could lead to substantial microbiota disruption and dysbiosis.^102^ Additionally, the type of antibiotics determines the composition of microflora and seems to correlate with GVHD incidence and outcomes. Antibiotics targeting anaerobic bacteria are associated with higher rates of GVHD-related mortality targeting the intestinal tract due to the development of mucus-degrading bacteria, but other organs such as skin and liver remained unaffected.^87^ The protective effect of antibiotics in HSCT could be explained by the reductions of inflammation and decreased radiation sensitivity in other murine models treated with antibiotics. Treatment with poorly absorbed ampicillin and non-absorbed neomycin reduced metabolic endotoxemia and inflammation in obese leptin-deficient mice (*ob/ob*) or mice fed with high-fat diet,^103^ indicating the benefit of antibiotic treatment on controlling obesity-associated “meta-inflammation”. More importantly, antibiotic treatment prevented bacterial translocation from the intestine into the bloodstream after radiation, a critical step for the activation of innate immune cells (e.g. dendritic cells) and cytokine storm triggering, therefore reducing gut permeability and tissue damage due to radiation sensitivity.^29,104^ These evidences provide different perspectives of how one might consider choosing the “right type” of antibiotics to preserve the beneficial bacteria taxa without compromising treatment efficacy.

Besides the type of antibiotics, timing of treatment also affects outcomes post-HSCT. Short-term discontinuous administration of ampicillin followed by a recovery period before HSCT resulted in worse survival outcomes in mice underwent allogeneic MHC mismatched HSCT.^84^ Continuous prophylactic treatment using broad-spectrum antibiotic cocktail with ampicillin, vancomycin, and neomycin demonstrated a protective effect in gut permeability and GVHD outcomes in high-fat diet fed obese mice.^50^ Short-term treatment with a similar antibiotic combination after HSCT also showed a complete protection from diarrhea, weight loss, and death in mice.^94^ However, there are evidences about prophylactic antibiotics resulted into microbiome disruption and higher transplant-related mortality than those who had antibiotics administered on or after day 0 or untreated patients indicating that more still needs to be delineated on the impact of different antibiotics as well as timing of administration.^102^

## Diet, prebiotics, and probiotics

Dietary intake has been shown to influence the component of the trillions of intestinal microorganisms.^70,71,105^ GVHD in the digestive tract has been shown to correlate with malnutrition, protein losing enteropathy, magnesium derangements, and deficiencies of zinc, vitamin B12 and vitamin D.^106^ Prebiotics are indigestible carbohydrates or substances metabolized by beneficial intestinal bacteria that help inhibiting the growth of other pathogenic bacteria, boosting the immune responses, and reducing the risk of various diseases.^107^ Short-chain fatty acids (SCFAs) are the product from the fermentation process of bacteria that recently been linked with prebiotics metabolism and health benefit.^108^ One of the most important SCFAs is butyrate, which has been shown to mitigate GVHD through the signaling by G-protein-coupled receptor 43 (GPR43) on the intestinal epithelial cells.^109^ Propionate and tryptophan has been shown to markedly increase in mice that survived long-term radiation exposure and administration of these metabolites also protected mice from cytokine storm and GI tract permeability.^90^ Dietary fiber changed the gut microbiota profiles by increasing diversity (Bacteroidaceae and Bifidobacteriaceae), which enhances SCFAs production.^110^ Microbiome changes also are dependent on the components of diets. The use of the “Western Diet,” which involves high fat and high sugar as opposed to only high fat, reduces Bacteroidetes and Actinobacteria, but increases Firmicutes, Proteobacteria, and Deferribacteres,^72^ while a high-fat only diet increases *Bacteroides, Enterobacteriaceae, Escherichia, Klebsiella*, and *Shigella*.^111^ With regard to diets, the length of time on the diet impacts outcome as does the composition of the diet with high-fat only diets ranging in the percent of fat (from 25–60%) in the diet.

Prebiotics consist of starch, fructans (inulin and fructo-oligosaccharides), and galacto-oligosaccharides that contribute to the increase of genus *Bifidobacterium* and *Lactobacilli* and the decrease of the genus *Bacteroides*^112^ which potentially beneficial for HSCT patients (https://doi.org/10.1101/2020.04.08.20058198) or cancer treatment with checkpoint blockade.^32^ Enteral supplementation of glutamine, fiber, and oligosaccharide in a retrospective study of allogeneic HSCT showed less mucositis grade 3–4, reduced weight loss and diarrhea, and improved survival percent at day 100 post-HSCT.^113^ In a recent article studying impact of pre-biotics and HSCT outcomes, resistant starch and prebiotics mixture (glutamine, polydextrose, and lactosucrose) were used pre-transplant and throughout 4 weeks post-HSCT. Patients that consumed prebiotics had reduced mucosal tissue damage and ameliorated acute GVHD.^114^

Probiotics are live microorganisms administered to improve health and have long been used as part of traditional diets. Supplementation of probiotics restores commensal flora in the intestine and has been shown to promote growth of beneficial species, thereby improving intestinal microbiome diversity and HSCT outcomes. In murine models, probiotics has been studied with some positive results. Oral administration of *Lactobacillus rhamnosus* GG in drinking water before and after HSCT resulted in reduced bacterial translocation, improved survival, and reduced acute GVHD pathogenesis.^115^ Administration of 17 butyrate-producing *Clostridia spp*. strains by oral gavage before and after allogeneic HSCT with an MHC mismatched model demonstrated a significant increase of butyrate in the intestine and better survival rate.^116^ A cocktail of *Clostridium bolteae, Ruminococcus gnavus, Ruminococcus torques*, and *Blautia producta* delivered by oral gavage also showed significant survival in mice.^117^ However, the efficacy of probiotics for HSCT in human is still questionable. The use of probiotics-enriched yogurt in a case report of a patient with autologous HSCT for treating mantle-cell lymphoma resulted in unexpected *Lactobacillus acidophilus* sepsis.^118^ In line with that, another clinical study reported the bloodstream infections with *Lactobacillus* bacteremia in patients with autologous and allogeneic HSCT within the first 100 d post-HSCT which implied that the toxicities from immunosuppression by conditioning regimens and mucosal disruption could contribute to bacteremia from probiotics consumption.^119^ In contrast, a study in children and adolescents undergoing allogeneic HSCT suggested that administration of probiotics with *Lactobacillus plantarum* was safe and feasible with no bacteremia case recorded.^120^

## Fecal microbiota transplantation

Aside from probiotics, another approach to introduce beneficial bacteria for HSCT patients for microbiome preservation or restoration is fecal microbiota transplantation (FMT). This is the method that is potentially applicable if patients have *Clostridium difficile* infections post-HSCT. Early clinical studies used FMT to treat patients with *Clostridium difficile* infections with high efficacy and minimal complications.^121–127^ FMT was also performed in steroid-resistant acute GVHD patients with no adverse effect and resulted in the restored microbiome diversity, an increase of peripheral regulatory T cells and better progression-free survival.^128–130^ Most recently, when FMT was used in patients with steroid-refractory or steroid-dependent intestinal GVHD, researchers observed a significant increase of microbiome alpha-diversity, increased abundances of butyrate-producing Clostridiales and *Blautia*.^131^ Taken together, FMT is a promising approach for intestinal GVHD patients and worth to be investigated further in a larger scale.

## Conclusions and future directions

Although microbiome characterization in HSCT studies has been investigated in the past decades, there are many questions remain unanswered regarding the “cause and effect” of specific bacterial taxa with the immune system, cell metabolism, and physiological consequences. Although obesity or high-fat diet consumption has been shown to affect microbiota profile and vice versa, little is known about whether microbiome changes in obesity, or high-fat diet exposure in either short-term or long-term could influence the HSCT outcomes. Most of the current concepts are descriptive and there is an urgent need on finding mechanism of how the different bacteria interact and how it affects immune parameters. The inbred laboratory mouse is still the cornerstone of preclinical HSCT modeling, but there is clearly a need to have it better reflect the human condition and involve human modifying factors such as obesity and prior infectious challenges, particularly in performing microbiome studies. This is also important with regard to finding a balance between having sufficient incorporation of the variables involved in human HSCT and also allowing for reproducibility of results. The gut pathology in the preclinical GVHD models does reflect the clinical scenario with common immune pathways and even similarities in microbiome content. Nonetheless, much more is needed to be known before targeted manipulation of the intestinal microbiome-immune system axis could be successfully applied as a therapeutic approach to limit HSCT complications such as GVHD and opportunistic infections, improve GVT effect and survival outcomes for patients. This not only applies to aGVHD affecting the gut but also in other organs and cGVHD given the linkage of the gut microbiome throughout every organ and immune parameter.

## References

[1] Thomas ED, Lochte HL Jr., Lu WC, Ferrebee JW. Intravenous infusion of bone marrow in patients receiving radiation and chemotherapy. N Engl J Med. 1957;257(11):491–17. doi:10.1056/NEJM195709122571102. 13464965

[2] Aschan J. Risk assessment in haematopoietic stem cell transplantation: conditioning. Best Pract Res Clin Haematol. 2007;20(2):295–310. doi:10.1016/j.beha.2006.09.004. 17448963

[3] Blazar BR, Murphy WJ, Abedi M. Advances in graft-versus-host disease biology and therapy. Nat Rev Immunol. 2012;12(6):443–458. doi:10.1038/nri3212. 22576252 PMC3552454

[4] Copelan EA. Hematopoietic stem-cell transplantation. N Engl J Med. 2006;354(17):1813–1826. doi:10.1056/NEJMra052638. 16641398

[5] Gyurkocza B, Sandmaier BM. Conditioning regimens for hematopoietic cell transplantation: one size does not fit all. Blood. 2014;124(3):344–353. doi:10.1182/blood-2014-02-514778. 24914142 PMC4102707

[6] Ogonek J, Kralj Juric M, Ghimire S, Varanasi PR, Holler E, Greinix H, Weissinger E. Immune reconstitution after allogeneic hematopoietic stem cell transplantation. Front Immunol. 2016;7:507. doi:10.3389/fimmu.2016.00507. 27909435 PMC5112259

[7] Akiyama H. [Infectious complications following hematopoietic stem cell transplantation]. [Rinsho Ketsueki] Jpn J Clin Hematol. 2008;49:576–582. 18800605

[8] Lyman GH, Kuderer NM, Djulbegovic B. Prophylactic granulocyte colony-stimulating factor in patients receiving dose-intensive cancer chemotherapy: a meta-analysis. Am J Med. 2002;112(5):406–411. doi:10.1016/s0002-9343(02)01036-7. 11904116

[9] Wingard JR, Elmongy M. Strategies for minimizing complications of neutropenia: prophylactic myeloid growth factors or antibiotics. Crit Rev Oncol Hematol. 2009;72(2):144–154. doi:10.1016/j.critrevonc.2009.01.003. 19237297

[10] Ferrara JL, Levine JE, Reddy P, Holler E. Graft-versus-host disease. Lancet. 2009;373(9674):1550–1561. doi:10.1016/S0140-6736(09)60237-3. 19282026 PMC2735047

[11] Zeiser R, Blazar BR. Pathophysiology of chronic graft-versus-host disease and therapeutic targets. N Engl J Med. 2017;377(26):2565–2579. doi:10.1056/NEJMra1703472. 29281578

[12] Zeiser R, Blazar BR. Acute graft-versus-host disease - biologic process, prevention, and therapy. N Engl J Med. 2017;377(22):2167–2179. doi:10.1056/NEJMra1609337. 29171820 PMC6034180

[13] Ferrara JL, Cooke KR, Teshima T. The pathophysiology of acute graft-versus-host disease. Int J Hematol. 2003;78(3):181–187. doi:10.1007/BF02983793. 14604275

[14] Zeiser R, Socie G, Blazar BR. Pathogenesis of acute graft-versus-host disease: from intestinal microbiota alterations to donor T cell activation. Br J Haematol. 2016;175(2):191–207. doi:10.1111/bjh.14295. 27619472

[15] Socie G, Blazar BR. Acute graft-versus-host disease: from the bench to the bedside. Blood. 2009;114(20):4327–4336. doi:10.1182/blood-2009-06-204669. 19713461 PMC2777122

[16] Weisdorf DJ, Snover DC, Haake R, Miller WJ, McGlave PB, Blazar B, Ramsay NK, Kersey JH, Filipovich A. Acute upper gastrointestinal graft-versus-host disease: clinical significance and response to immunosuppressive therapy. Blood. 1990;76(3):624–629. 2378989

[17] Yeh SP, Liao YM, Hsu CH, Chen CL, Shen YC, Hsueh CT, Huang HH, Lin CL, Chiu CF. Gastric bleeding due to graft-vs-host disease: discrepancy between endoscopic and histologic assessment. Am J Clin Pathol. 2004;122(6):919–925. doi:10.1309/23DA-L9F6-P74X-WJHL. 15539384

[18] Wu D, Hockenbery DM, Brentnall TA, Baehr PH, Ponec RJ, Kuver R, Tzung SP, Todaro JL, McDonald GB. Persistent nausea and anorexia after marrow transplantation: a prospective study of 78 patients. Transplantation. 1998;66(10):1319–1324. doi:10.1097/00007890-199811270-00010. 9846516

[19] Naymagon S, Naymagon L, Wong SY, Ko HM, Renteria A, Levine J, Colombel JF, Ferrara J. Acute graft-versus-host disease of the gut: considerations for the gastroenterologist. Nat Rev Gastroenterol Hepatol. 2017;14(12):711–726. doi:10.1038/nrgastro.2017.126. 28951581 PMC6240460

[20] Kirkpatrick ID, Greenberg HM. Gastrointestinal complications in the neutropenic patient: characterization and differentiation with abdominal CT. Radiology. 2003;226(3):668–674. doi:10.1148/radiol.2263011932. 12601214

[21] Mahgerefteh SY, Sosna J, Bogot N, Shapira MY, Pappo O, Bloom AI. Radiologic imaging and intervention for gastrointestinal and hepatic complications of hematopoietic stem cell transplantation. Radiology. 2011;258(3):660–671. doi:10.1148/radiol.10100025. 21339345

[22] Budjan J, Michaely HJ, Attenberger U, Haneder S, Heidenreich D, Kreil S, Nolte F, Hofmann WK, Schoenberg SO, Klein SA. Assessment of acute intestinal graft versus host disease by abdominal magnetic resonance imaging at 3 Tesla. Eur Radiol. 2014;24(8):1835–1844. doi:10.1007/s00330-014-3224-8. 24863887

[23] Blazar BR, Hill GR, Murphy WJ. Dissecting the biology of allogeneic HSCT to enhance the GvT effect whilst minimizing GvHD. Nat Rev Clin Oncol. 2020;17(8):475–492. doi:10.1038/s41571-020-0356-4. 32313224 PMC7901860

[24] Weiden PL, Flournoy N, Thomas ED, Prentice R, Fefer A, Buckner CD, Storb R. Antileukemic effect of graft-versus-host disease in human recipients of allogeneic-marrow grafts. N Engl J Med. 1979;300(19):1068–1073. doi:10.1056/NEJM197905103001902. 34792

[25] Gattinoni L, Finkelstein SE, Klebanoff CA, Antony PA, Palmer DC, Spiess PJ, Hwang LN, Yu Z, Wrzesinski C, Heimann DM, et al. Removal of homeostatic cytokine sinks by lymphodepletion enhances the efficacy of adoptively transferred tumor-specific CD8+ T cells. J Exp Med. 2005;202(7):907–912. doi:10.1084/jem.20050732. 16203864 PMC1397916

[26] Muranski P, Boni A, Wrzesinski C, Citrin DE, Rosenberg SA, Childs R, Restifo NP. Increased intensity lymphodepletion and adoptive immunotherapy--how far can we go? Nat Clin Pract Oncol. 2006;3(12):668–681. doi:10.1038/ncponc0666. 17139318 PMC1773008

[27] Kochenderfer JN, Somerville RP, Lu T, Shi V, Bot A, Rossi J, Xue A, Goff SL, Yang JC, Sherry RM, Klebanoff CA, et al. Lymphoma remissions caused by anti-CD19 chimeric antigen receptor T cells are associated with high serum interleukin-15 levels. J Clin Oncol. 2017;35(16):1803–1813. doi:10.1200/JCO.2016.71.3024. 28291388 PMC5455597

[28] Turtle CJ, Hanafi LA, Berger C, Hudecek M, Pender B, Robinson E, Hawkins R, Chaney C, Cherian S, Chen X, Soma L, Wood B, Li D, Heimfeld S, Riddell SA, Maloney DG. Immunotherapy of non-Hodgkin’s lymphoma with a defined ratio of CD8+ and CD4+ CD19-specific chimeric antigen receptor-modified T cells. Sci Transl Med. 2016;8(355):355ra116. doi:10.1126/scitranslmed.aaf8621. PMC5045301 27605551

[29] Paulos CM, Wrzesinski C, Kaiser A, Hinrichs CS, Chieppa M, Cassard L, Palmer DC, Boni A, Muranski P, Yu Z, Gattinoni L, Antony PA, Rosenberg SA, Restifo NP. Microbial translocation augments the function of adoptively transferred self/tumor-specific CD8+ T cells via TLR4 signaling. J Clin Invest. 2007;117(8):2197–2204. doi:10.1172/JCI32205. 17657310 PMC1924500

[30] Iida N, Dzutsev A, Stewart CA, Smith L, Bouladoux N, Weingarten RA, Molina DA, Salcedo R, Back T, Cramer S, Dai RM, Kiu H, Cardone M, Naik S, Patri AK, Wang E, Marincola FM, Frank KM, Belkaid Y, Trinchieri G, Goldszmid RS. Commensal bacteria control cancer response to therapy by modulating the tumor microenvironment. Science. 2013;342(6161):967–970. doi:10.1126/science.1240527. 24264989 PMC6709532

[31] Viaud S, Saccheri F, Mignot G, Yamazaki T, Daillère R, Hannani D, Enot DP, Pfirschke C, Engblom C, Pittet MJ, et al. The intestinal microbiota modulates the anticancer immune effects of cyclophosphamide. Science. 2013;342(6161):971–976. doi:10.1126/science.1240537. 24264990 PMC4048947

[32] Sivan A, Corrales L, Hubert N, Williams JB, Aquino-Michaels K, Earley ZM, Benyamin FW, Lei YM, Jabri B, Alegre ML, Chang EB, Gajewski TF. Commensal bifidobacterium promotes antitumor immunity and facilitates anti-PD-L1 efficacy. Science. 2015;350(6264):1084–1089. doi:10.1126/science.aac4255. 26541606 PMC4873287

[33] Vetizou M, Pitt JM, Daillere R, Lepage P, Waldschmitt N, Flament C, Rusakiewicz S, Routy B, Roberti MP, Duong CP, et al. Anticancer immunotherapy by CTLA-4 blockade relies on the gut microbiota. Science. 2015;350(6264):1079–1084. doi:10.1126/science.aad1329. 26541610 PMC4721659

[34] Mazmanian SK, Liu CH, Tzianabos AO, Kasper DL. An immunomodulatory molecule of symbiotic bacteria directs maturation of the host immune system. Cell. 2005;122(1):107–118. doi:10.1016/j.cell.2005.05.007. 16009137

[35] Round JL, Mazmanian SK. The gut microbiota shapes intestinal immune responses during health and disease. Nat Rev Immunol. 2009;9(5):313–323. doi:10.1038/nri2515. 19343057 PMC4095778

[36] Lavin R, DiBenedetto N, Yeliseyev V, Delaney M, Gnotobiotic BL. Conventional mouse systems to support microbiota based studies. Curr Protoc Immunol. 2018;121(1):e48. doi:10.1002/cpim.48. 30008984 PMC6040836

[37] Park JC, Im SH. Of men in mice: the development and application of a humanized gnotobiotic mouse model for microbiome therapeutics. Exp Mol Med. 2020;52(9):1383–1396. doi:10.1038/s12276-020-0473-2. 32908211 PMC8080820

[38] Mallapaty S. Gnotobiotics: getting a grip on the microbiome boom. Lab Anim (NY). 2017;46(10):373–377. doi:10.1038/laban.1344. 28984861

[39] Beura LK, Hamilton SE, Bi K, Schenkel JM, Odumade OA, Casey KA, Thompson EA, Fraser KA, Rosato PC, Filali-Mouhim A, et al. Normalizing the environment recapitulates adult human immune traits in laboratory mice. Nature. 2016;532(7600):512–516. doi:10.1038/nature17655. 27096360 PMC4871315

[40] Nishimoto S, Fukuda D, Higashikuni Y, Tanaka K, Hirata Y, Murata C, Kim-Kaneyama JR, Sato F, Bando M, Yagi S, et al. Obesity-induced DNA released from adipocytes stimulates chronic adipose tissue inflammation and insulin resistance. Sci Adv. 2016;2(3):e1501332. doi:10.1126/sciadv.1501332. 27051864 PMC4820373

[41] Franceschi C, Garagnani P, Parini P, Giuliani C, Santoro A. Inflammaging: a new immune-metabolic viewpoint for age-related diseases. Nat Rev Endocrinol. 2018;14(10):576–590. doi:10.1038/s41574-018-0059-4. 30046148

[42] Thaiss CA, Levy M, Grosheva I, Zheng D, Soffer E, Blacher E, Braverman S, Tengeler AC, Barak O, Elazar M, et al. Hyperglycemia drives intestinal barrier dysfunction and risk for enteric infection. Science. 2018;359(6382):1376–1383. doi:10.1126/science.aar3318. 29519916

[43] Thevaranjan N, Puchta A, Schulz C, Naidoo A, Szamosi JC, Verschoor CP, Loukov D, Schenck LP, Jury J, Foley KP, et al. Age-associated microbial dysbiosis promotes intestinal permeability, systemic inflammation, and macrophage dysfunction. Cell Host Microbe. 2017;21(4):455–466 e454. doi:10.1016/j.chom.2017.03.002. 28407483 PMC5392495

[44] Zhao Y, Liu Q, Yang L, He D, Wang L, Tian J, Li Y, Zi F, Bao H, Yang Y, Zheng Y, Shi J, Xue X, Cai Z. TLR4 inactivation protects from graft-versus-host disease after allogeneic hematopoietic stem cell transplantation. Cell Mol Immunol. 2013;10(2):165–175. doi:10.1038/cmi.2012.58. 23262974 PMC4003043

[45] Nathan C, Ding A. Nonresolving inflammation. Cell. 2010;140(6):871–882. doi:10.1016/j.cell.2010.02.029. 20303877

[46] Gregor MF, Hotamisligil GS. Inflammatory mechanisms in obesity. Annu Rev Immunol. 2011;29:415–445. doi:10.1146/annurev-immunol-031210-101322. 21219177

[47] Lumeng CN, Bodzin JL, Saltiel AR. Obesity induces a phenotypic switch in adipose tissue macrophage polarization. J Clin Invest. 2007;117(1):175–184. doi:10.1172/JCI29881. 17200717 PMC1716210

[48] Berg AH, Scherer PE. Adipose tissue, inflammation, and cardiovascular disease. Circ Res. 2005;96(9):939–949. doi:10.1161/01.RES.0000163635.62927.34. 15890981

[49] Hamilton MK, Ronveaux CC, Rust BM, Newman JW, Hawley M, Barile D, Mills DA, Raybould HE. Prebiotic milk oligosaccharides prevent development of obese phenotype, impairment of gut permeability, and microbial dysbiosis in high fat-fed mice. Am J Physiol Gastrointest Liver Physiol. 2017;312(5):G474–G487. doi:10.1152/ajpgi.00427.2016. 28280143 PMC5451559

[50] Khuat LT, Le CT, Pai CS, Shields-Cutler RR, Holtan SG, Rashidi A, Parker SL, Knights D, Luna JI, Dunai C, et al. Obesity induces gut microbiota alterations and augments acute graft-versus-host disease after allogeneic stem cell transplantation. Sci Transl Med. 2020;12:571. doi:10.1126/scitranslmed.aay7713. PMC8525601 33239390

[51] Fuji S, Kim SW, Yoshimura K, Akiyama H, Okamoto S, Sao H, Takita J, Kobayashi N, Mori S. Possible association between obesity and posttransplantation complications including infectious diseases and acute graft-versus-host disease. Biol Blood Marrow Transplant. 2009;15(1):73–82. doi:10.1016/j.bbmt.2008.10.029. 19135945

[52] Jaime-Perez JC, Colunga-Pedraza PR, Gutierrez-Gurrola B, Brito-Ramirez AS, Gutierrez-Aguirre H, Cantu-Rodriguez OG, Herrera-Garza JL, Gomez-Almaguer D. Obesity is associated with higher overall survival in patients undergoing an outpatient reduced-intensity conditioning hematopoietic stem cell transplant. Blood Cells Mol Dis. 2013;51(1):61–65. doi:10.1016/j.bcmd.2013.01.010. 23422842

[53] Navarro WH, Loberiza FR, Jr., Bajorunaite R, van Besien K, Vose JM, Lazarus HM, Rizzo JD. Effect of body mass index on mortality of patients with lymphoma undergoing autologous hematopoietic cell transplantation. Biol Blood Marrow Transplant. 2006;12(5):541–551. doi:10.1016/j.bbmt.2005.12.033. 16635789

[54] Bluher M. The distinction of metabolically ‘healthy’ from ‘unhealthy’ obese individuals. Curr Opin Lipidol. 2010;21(1):38–43. doi:10.1097/MOL.0b013e3283346ccc. 19915462

[55] O’Toole PW, Jeffery IB. Gut microbiota and aging. Science. 2015;350(6265):1214–1215. doi:10.1126/science.aac8469. 26785481

[56] Claesson MJ, Jeffery IB, Conde S, Power SE, O'Connor EM, Cusack S, Harris HM, Coakley M, Lakshminarayanan B, O'Sullivan O, et al. Gut microbiota composition correlates with diet and health in the elderly. Nature. 2012;488(7410):178–184. doi:10.1038/nature11319. 22797518

[57] Ley RE, Backhed F, Turnbaugh P, Lozupone CA, Knight RD, Gordon JI. Obesity alters gut microbial ecology. Proc Natl Acad Sci U S A. 2005;102(31):11070–11075. doi:10.1073/pnas.0504978102. 16033867 PMC1176910

[58] Turnbaugh PJ, Hamady M, Yatsunenko T, Cantarel BL, Duncan A, Ley RE, Sogin ML, Jones WJ, Roe BA, Affourtit JP, et al. A core gut microbiome in obese and lean twins. Nature. 2009;457(7228):480–484. doi:10.1038/nature07540. 19043404 PMC2677729

[59] Liu C, Frank DN, Horch M, Chau S, Ir D, Horch EA, Tretina K, van Besien K, Lozupone CA, Nguyen VH. Associations between acute gastrointestinal GvHD and the baseline gut microbiota of allogeneic hematopoietic stem cell transplant recipients and donors. Bone Marrow Transplant. 2017;52(12):1643–1650. doi:10.1038/bmt.2017.200. 28967895

[60] Taur Y, Jenq RR, Perales MA, Littmann ER, Morjaria S, Ling L, No D, Gobourne A, Viale A, Dahi PB, Ponce DM, et al. The effects of intestinal tract bacterial diversity on mortality following allogeneic hematopoietic stem cell transplantation. Blood. 2014;124(7):1174–1182. doi:10.1182/blood-2014-02-554725. 24939656 PMC4133489

[61] Malard F, Gasc C, Plantamura E, Dore J. High gastrointestinal microbial diversity and clinical outcome in graft-versus-host disease patients. Bone Marrow Transplant. 2018. doi:10.1038/s41409-018-0254-x. PMC6281565 29904128

[62] Wilmanski T, Diener C, Rappaport N, Patwardhan S, Wiedrick J, Lapidus J, Earls JC, Zimmer A, Glusman G, Robinson M, Yurkovich JT, et al. Gut microbiome pattern reflects healthy ageing and predicts survival in humans. Nat Metab. 2021;3(2):274–286. doi:10.1038/s42255-021-00348-0. 33619379 PMC8169080

[63] Komura T, Ikeda T, Yasui C, Saeki S, Nishikawa Y. Mechanism underlying prolongevity induced by bifidobacteria in Caenorhabditis elegans. Biogerontology. 2013;14(1):73–87. doi:10.1007/s10522-012-9411-6. 23291976

[64] Goodrich JK, Waters JL, Poole AC, Sutter JL, Koren O, Blekhman R, Beaumont M, Van Treuren W, Knight R, Bell JT, Spector TD, Clark AG, Ley RE. Human genetics shape the gut microbiome. Cell. 2014;159(4):789–799. doi:10.1016/j.cell.2014.09.053. 25417156 PMC4255478

[65] Weiss BM, Vogl DT, Berger NA, Stadtmauer EA, Lazarus HM. Trimming the fat: obesity and hematopoietic cell transplantation. Bone Marrow Transplant. 2013;48(9):1152–1160. doi:10.1038/bmt.2012.201. 23103679

[66] Pine M, Wang L, Harrell FE, Jr., Calder C, Manes B, Evans M, Domm J, Frangoul H. The effect of obesity on outcome of unrelated cord blood transplant in children with malignant diseases. Bone Marrow Transplant. 2011;46(10):1309–1313. doi:10.1038/bmt.2010.312. 21151185

[67] Ma BW, Bokulich NA, Castillo PA, Kananurak A, Underwood MA, Mills DA, Bevins CL. Routine habitat change: a source of unrecognized transient alteration of intestinal microbiota in laboratory mice. PLoS One. 2012;7(10):e47416. doi:10.1371/journal.pone.0047416. 23082164 PMC3474821

[68] Sofi MH, Gudi R, Karumuthil-Melethil S, Perez N, Johnson BM, Vasu C. pH of drinking water influences the composition of gut microbiome and type 1 diabetes incidence. Diabetes. 2014;63(2):632–644. doi:10.2337/db13-0981. 24194504 PMC3900548

[69] Bloomfield SF, Rook GA, Scott EA, Shanahan F, Stanwell-Smith R, Turner P. Time to abandon the hygiene hypothesis: new perspectives on allergic disease, the human microbiome, infectious disease prevention and the role of targeted hygiene. Perspect Public Health. 2016;136(4):213–224. doi:10.1177/1757913916650225. 27354505 PMC4966430

[70] Turnbaugh PJ, Backhed F, Fulton L, Gordon JI. Diet-induced obesity is linked to marked but reversible alterations in the mouse distal gut microbiome. Cell Host Microbe. 2008;3(4):213–223. doi:10.1016/j.chom.2008.02.015. 18407065 PMC3687783

[71] Ooi JH, Waddell A, Lin YD, Albert I, Rust LT, Holden V, Cantorna MT. Dominant effects of the diet on the microbiome and the local and systemic immune response in mice. PLoS One. 2014;9(1):e86366. doi:10.1371/journal.pone.0086366. 24489720 PMC3906035

[72] Jena PK, Sheng L, Di Lucente J, Jin LW, Maezawa I, Wan YY. Dysregulated bile acid synthesis and dysbiosis are implicated in Western diet-induced systemic inflammation, microglial activation, and reduced neuroplasticity. FASEB J. 2018;32(5):2866–2877. doi:10.1096/fj.201700984RR. 29401580 PMC5901391

[73] De Angelis M, Ferrocino I, Calabrese FM, De Filippis F, Cavallo N, Siragusa S, Rampelli S, Di Cagno R, Rantsiou K, Vannini L, Pellegrini N, et al. Diet influences the functions of the human intestinal microbiome. Sci Rep. 2020;10(1):4247. doi:10.1038/s41598-020-61192-y. 32144387 PMC7060259

[74] Miller WP, Srinivasan S, Panoskaltsis-Mortari A, Singh K, Sen S, Hamby K, Deane T, Stempora L, Beus J, et al. GVHD after haploidentical transplantation: a novel, MHC-defined rhesus macaque model identifies CD28- CD8+ T cells as a reservoir of breakthrough T-cell proliferation during costimulation blockade and sirolimus-based immunosuppression. Blood. 2010;116(24):5403–5418. doi:10.1182/blood-2010-06-289272. 20833977 PMC3012549

[75] Watkins BK, Tkachev V, Furlan SN, Hunt DJ, Betz K, Yu A, Brown M, Poirier N, Zheng HB, Taraseviciute A, et al. CD28 blockade controls T cell activation to prevent graft-versus-host disease in primates. J Clin Invest. 2018;128(9):3991–4007. doi:10.1172/JCI98793. 30102255 PMC6118599

[76] Tkachev V, Kaminski J, Potter EL, Furlan SN, Yu A, Hunt DJ, McGuckin C, Zheng H, Colonna L, Gerdemann U, et al. Spatiotemporal single-cell profiling reveals that invasive and tissue-resident memory donor CD8(+) T cells drive gastrointestinal acute graft-versus-host disease. Sci Transl Med. 2021;13: 576. doi:10.1126/scitranslmed.abc0227. PMC9469805 33441422

[77] Vrecenak JD, Pearson EG, Todorow CA, Li H, Johnson MP, Flake AW. Preclinical canine model of graft-versus-host disease after in utero hematopoietic cell transplantation. Biol Blood Marrow Transplant. 2018;24(9):1795–1801. doi:10.1016/j.bbmt.2018.05.020. 29802901

[78] Kolb H, Sale GE, Lerner KG, Storb R, Thomas ED. Pathology of acute graft-versus-host disease in the dog. An autopsy study of ninety-five dogs. Am J Pathol. 1979;96:581–594. 38670 PMC2042451

[79] Weisburg WG, Barns SM, Pelletier DA, Lane DJ. 16S ribosomal DNA amplification for phylogenetic study. J Bacteriol. 1991;173(2):697–703. doi:10.1128/jb.173.2.697-703.1991. 1987160 PMC207061

[80] Caporaso JG, Lauber CL, Walters WA, Berg-Lyons D, Huntley J, Fierer N, Owens SM, Betley J, Fraser L, Bauer M, Gormley N, Gilbert JA, Smith G, Knight R. Ultra-high-throughput microbial community analysis on the Illumina HiSeq and MiSeq platforms. ISME J. 2012;6(8):1621–1624. doi:10.1038/ismej.2012.8. 22402401 PMC3400413

[81] Jovel J, Patterson J, Wang W, Hotte N, O'Keefe S, Mitchel T, Perry T, Kao D, Mason AL, Madsen KL, Wong GK. Characterization of the gut microbiome using 16s or shotgun metagenomics. Front Microbiol. 2016;7:459. doi:10.3389/fmicb.2016.00459. 27148170 PMC4837688

[82] Loman NJ, Constantinidou C, Chan JZ, Halachev M, Sergeant M, Penn CW, Robinson ER, Pallen MJ. High-throughput bacterial genome sequencing: an embarrassment of choice, a world of opportunity. Nat Rev Microbiol. 2012;10(9):599–606. doi:10.1038/nrmicro2850. 22864262

[83] Holler E, Butzhammer P, Schmid K, Hundsrucker C, Koestler J, Peter K, Zhu W, Sporrer D, Hehlgans T, et al. Metagenomic analysis of the stool microbiome in patients receiving allogeneic stem cell transplantation: loss of diversity is associated with use of systemic antibiotics and more pronounced in gastrointestinal graft-versus-host disease. Biol Blood Marrow Transplant. 2014;20(5):640–645. doi:10.1016/j.bbmt.2014.01.030. 24492144 PMC4973578

[84] Jenq RR, Ubeda C, Taur Y, Menezes CC, Khanin R, Dudakov JA, Liu C, West ML, Singer NV, Equinda MJ, et al. Regulation of intestinal inflammation by microbiota following allogeneic bone marrow transplantation. J Exp Med. 2012;209(5):903–911. doi:10.1084/jem.20112408. 22547653 PMC3348096

[85] Candela M, Biagi E, Maccaferri S, Turroni S, Brigidi P. Intestinal microbiota is a plastic factor responding to environmental changes. Trends Microbiol. 2012;20(8):385–391. doi:10.1016/j.tim.2012.05.003. 22672911

[86] Claesson MJ, Clooney AG, O’Toole PW. A clinician’s guide to microbiome analysis. Nat Rev Gastroenterol Hepatol. 2017;14(10):585–595. doi:10.1038/nrgastro.2017.97. 28790452

[87] Shono Y, Docampo MD, Peled JU, Perobelli SM, Velardi E, Tsai JJ, Slingerland AE, Smith OM, Young LF, et al. Increased GVHD-related mortality with broad-spectrum antibiotic use after allogeneic hematopoietic stem cell transplantation in human patients and mice. Sci Transl Med. 2016;8(339):339ra371. doi:10.1126/scitranslmed.aaf2311. PMC4991773 27194729

[88] Jenq RR, Taur Y, Devlin SM, Ponce DM, Goldberg JD, Ahr KF, Littmann ER, Ling L, Gobourne AC, Miller LC, et al. Intestinal blautia is associated with reduced death from graft-versus-host disease. Biol Blood Marrow Transplant. 2015;21(8):1373–1383. doi:10.1016/j.bbmt.2015.04.016. 25977230 PMC4516127

[89] Biagi E, Zama D, Nastasi C, Consolandi C, Fiori J, Rampelli S, Turroni S, Centanni M, Severgnini M, Peano C, et al. Gut microbiota trajectory in pediatric patients undergoing hematopoietic SCT. Bone Marrow Transplant. 2015;50(7):992–998. doi:10.1038/bmt.2015.16. 25893458

[90] Guo H, Chou WC, Lai Y, Liang K, Tam JW, Brickey WJ, Chen L, Montgomery ND, Li X, Bohannon LM, et al. Multi-omics analyses of radiation survivors identify radioprotective microbes and metabolites. Science. 2020;370: 6516. doi:10.1126/science.aay9097. PMC7898465 33122357

[91] Blaser MJ. The microbiome revolution. J Clin Invest. 2014;124(10):4162–4165. doi:10.1172/JCI78366. 25271724 PMC4191014

[92] Van Bekkum DW, Knaan S. Role of bacterial microflora in development of intestinal lesions from graft-versus-host reaction. J Natl Cancer Inst. 1977;58:787–790. 14265 10.1093/jnci/58.3.787

[93] Jones JM, Wilson R, Bealmear PM. Mortality and gross pathology of secondary disease in germfree mouse radiation chimeras. Radiat Res. 1971;45:577–588. 4396814

[94] Song Q, Wang X, Wu X, Kang TH, Qin H, Zhao D, Jenq RR, van den Brink MRM, Riggs AD, Martin PJ, Chen YZ, Zeng D. IL-22-dependent dysbiosis and mononuclear phagocyte depletion contribute to steroid-resistant gut graft-versus-host disease in mice. Nat Commun. 2021;12(1):805. doi:10.1038/s41467-021-21133-3. 33547295 PMC7865028

[95] Koyama M, Mukhopadhyay P, Schuster IS, Henden AS, Hulsdunker J, Varelias A, Vetizou M, Kuns RD, Robb RJ, Zhang P, et al. MHC class II antigen presentation by the intestinal epithelium initiates graft-versus-host disease and is influenced by the microbiota. Immunity. 2019;51(5):885–898 e887. doi:10.1016/j.immuni.2019.08.011. 31542340 PMC6959419

[96] Hulsdunker J, Ottmuller KJ, Neeff HP, Koyama M, Gao Z, Thomas OS, Follo M, Al-Ahmad A, Prinz G, Duquesne S, et al. Neutrophils provide cellular communication between ileum and mesenteric lymph nodes at graft-versus-host disease onset. Blood. 2018;131(16):1858–1869. doi:10.1182/blood-2017-10-812891. 29463561 PMC5909763

[97] Almeida L, Dhillon-LaBrooy A, Castro CN, Adossa N, Carriche GM, Guderian M, Lippens S, Dennerlein S, Hesse C, Lambrecht BN, et al. Ribosome-targeting antibiotics impair T cell effector function and ameliorate autoimmunity by blocking mitochondrial protein synthesis. Immunity. 2021;54(1):68–83 e66. doi:10.1016/j.immuni.2020.11.001. 33238133 PMC7837214

[98] Taur Y, Coyte K, Schluter J, Robilotti E, Figueroa C, Gjonbalaj M, Littmann ER, Ling L, Miller L, Gyaltshen Y, et al. Reconstitution of the gut microbiota of antibiotic-treated patients by autologous fecal microbiota transplant. Sci Transl Med. 2018;10(460). doi:10.1126/scitranslmed.aap9489 PMC6468978 30257956

[99] Storb R, Prentice RL, Buckner CD, Clift RA, Appelbaum F, Deeg J, Doney K, Hansen JA, Mason M, Sanders JE, et al. Graft-versus-host disease and survival in patients with aplastic anemia treated by marrow grafts from HLA-identical siblings. Beneficial effect of a protective environment. N Engl J Med. 1983;308(6):302–307. doi:10.1056/NEJM198302103080602. 6337323

[100] Vossen JM, Heidt PJ, Van Den Berg H, Gerritsen EJ, Hermans J, Dooren LJ. Prevention of infection and graft-versus-host disease by suppression of intestinal microflora in children treated with allogeneic bone marrow transplantation. Eur J Clin Microbiol Infect Dis. 1990;9(1):14–23. doi:10.1007/BF01969527. 2105890

[101] Vossen JM, Guiot HF, Lankester AC, Vossen AC, Bredius RG, Wolterbeek R, Bakker HD, Heidt PJ. Complete suppression of the gut microbiome prevents acute graft-versus-host disease following allogeneic bone marrow transplantation. PLoS One. 2014;9(9):e105706. doi:10.1371/journal.pone.0105706. 25180821 PMC4152127

[102] Weber D, Jenq RR, Peled JU, Taur Y, Hiergeist A, Koestler J, Dettmer K, Weber M, Wolff D, Hahn J, et al. Microbiota disruption induced by early use of broad-spectrum antibiotics is an independent risk factor of outcome after allogeneic stem cell transplantation. Biol Blood Marrow Transplant. 2017;23(5):845–852. doi:10.1016/j.bbmt.2017.02.006. 28232086 PMC5546237

[103] Cani PD, Bibiloni R, Knauf C, Waget A, Neyrinck AM, Delzenne NM, Burcelin R. Changes in gut microbiota control metabolic endotoxemia-induced inflammation in high-fat diet-induced obesity and diabetes in mice. Diabetes. 2008;57(6):1470–1481. doi:10.2337/db07-1403. 18305141

[104] Espinosa-Carrasco G, Villard M, Le Saout C, Louis-Plence P, Vicente R, Hernandez J. Systemic LPS translocation activates cross-presenting dendritic cells but is dispensable for the breakdown of CD8+ T cell peripheral tolerance in irradiated mice. PLoS One. 2015;10(6):e0130041. doi:10.1371/journal.pone.0130041. 26075613 PMC4468093

[105] David LA, Maurice CF, Carmody RN, Gootenberg DB, Button JE, Wolfe BE, Ling AV, Devlin AS, Varma Y, Fischbach MA, et al. Diet rapidly and reproducibly alters the human gut microbiome. Nature. 2014;505(7484):559–563. doi:10.1038/nature12820. 24336217 PMC3957428

[106] van der Meij BS, de Graaf P, Wierdsma NJ, Langius JA, Janssen JJ, van Leeuwen PA, Visser OJ. Nutritional support in patients with GVHD of the digestive tract: state of the art. Bone Marrow Transplant. 2013;48(4):474–482. doi:10.1038/bmt.2012.124. 22773121

[107] Bindels LB, Delzenne NM, Cani PD, Walter J. Towards a more comprehensive concept for prebiotics. Nat Rev Gastroenterol Hepatol. 2015;12(5):303–310. doi:10.1038/nrgastro.2015.47. 25824997

[108] Gibson GR, Hutkins R, Sanders ME, Prescott SL, Reimer RA, Salminen SJ, Scott K, Stanton C, Swanson KS, Cani PD, et al. Expert consensus document: the International Scientific Association for Probiotics and Prebiotics (ISAPP) consensus statement on the definition and scope of prebiotics. Nat Rev Gastroenterol Hepatol. 2017;14(8):491–502. doi:10.1038/nrgastro.2017.75. 28611480

[109] Fujiwara H, Docampo MD, Riwes M, Peltier D, Toubai T, Henig I, Wu SJ, Kim S, Taylor A, Brabbs S, Liu C, et al. Microbial metabolite sensor GPR43 controls severity of experimental GVHD. Nat Commun. 2018;9(1):3674. doi:10.1038/s41467-018-06048-w. 30201970 PMC6131147

[110] Trompette A, Gollwitzer ES, Yadava K, Sichelstiel AK, Sprenger N, Ngom-Bru C, Blanchard C, Junt T, Nicod LP, Harris NL, Marsland BJ. Gut microbiota metabolism of dietary fiber influences allergic airway disease and hematopoiesis. Nat Med. 2014;20(2):159–166. doi:10.1038/nm.3444. 24390308

[111] Singh RP, Halaka DA, Hayouka Z, Tirosh O. High-fat diet induced alteration of mice microbiota and the functional ability to utilize fructooligosaccharide for ethanol production. Front Cell Infect Microbiol. 2020;10:376. doi:10.3389/fcimb.2020.00376. 32850478 PMC7426704

[112] Gibson GR, Beatty ER, Wang X, Cummings JH. Selective stimulation of bifidobacteria in the human colon by oligofructose and inulin. Gastroenterology. 1995;108(4):975–982. doi:10.1016/0016-5085(95)90192-2. 7698613

[113] Iyama S, Sato T, Tatsumi H, Hashimoto A, Tatekoshi A, Kamihara Y, Horiguchi H, Ibata S, Ono K, Murase K, et al. Efficacy of enteral supplementation enriched with glutamine, fiber, and oligosaccharide on mucosal injury following hematopoietic stem cell transplantation. Case Rep Oncol. 2014;7(3):692–699. doi:10.1159/000368714. 25493082 PMC4255989

[114] Yoshifuji K, Inamoto K, Kiridoshi Y, Takeshita K, Sasajima S, Shiraishi Y, Yamashita Y, Nisaka Y, Ogura Y, Takeuchi R, et al. Prebiotics protect against acute graft-versus-host disease and preserve the gut microbiota in stem cell transplantation. Blood Adv. 2020;4(19):4607–4617. doi:10.1182/bloodadvances.2020002604. 32991720 PMC7556149

[115] Gerbitz A, Schultz M, Wilke A, Linde HJ, Scholmerich J, Andreesen R, Holler E. Probiotic effects on experimental graft-versus-host disease: let them eat yogurt. Blood. 2004;103(11):4365–4367. doi:10.1182/blood-2003-11-3769. 14962899

[116] Mathewson ND, Jenq R, Mathew AV, Koenigsknecht M, Hanash A, Toubai T, Oravecz-Wilson K, Wu SR, Sun Y, Rossi C, et al. Gut microbiome-derived metabolites modulate intestinal epithelial cell damage and mitigate graft-versus-host disease. Nat Immunol. 2016;17(5):505–513. doi:10.1038/ni.3400. 26998764 PMC4836986

[117] Simms-Waldrip TR, Sunkersett G, Coughlin LA, Savani MR, Arana C, Kim J, Kim M, Zhan X, Greenberg DE, Xie Y, et al. Antibiotic-induced depletion of anti-inflammatory clostridia is associated with the development of graft-versus-host disease in pediatric stem cell transplantation Patients. Biol Blood Marrow Transplant. 2017;23(5):820–829. doi:10.1016/j.bbmt.2017.02.004. 28192251

[118] Mehta A, Rangarajan S, Borate U. A cautionary tale for probiotic use in hematopoietic SCT patients-Lactobacillus acidophilus sepsis in a patient with mantle cell lymphoma undergoing hematopoietic SCT. Bone Marrow Transplant. 2013;48(3):461–462. doi:10.1038/bmt.2012.153. 22890287

[119] Cohen SA, Woodfield MC, Boyle N, Stednick Z, Boeckh M, Pergam SA. Incidence and outcomes of bloodstream infections among hematopoietic cell transplant recipients from species commonly reported to be in over-the-counter probiotic formulations. Transpl Infect Dis. 2016;18(5):699–705. doi:10.1111/tid.12587. 27501401 PMC5160044

[120] Ladas EJ, Bhatia M, Chen L, Sandler E, Petrovic A, Berman DM, Hamblin F, Gates M, Hawks R, Sung L, Nieder M. The safety and feasibility of probiotics in children and adolescents undergoing hematopoietic cell transplantation. Bone Marrow Transplant. 2016;51(2):262–266. doi:10.1038/bmt.2015.275. 26569091

[121] Gough E, Shaikh H, Manges AR. Systematic review of intestinal microbiota transplantation (fecal bacteriotherapy) for recurrent Clostridium difficile infection. Clin Infect Dis. 2011;53(10):994–1002. doi:10.1093/cid/cir632. 22002980

[122] Kassam Z, Lee CH, Yuan Y, Hunt RH. Fecal microbiota transplantation for Clostridium difficile infection: systematic review and meta-analysis. Am J Gastroenterol. 2013;108(4):500–508. doi:10.1038/ajg.2013.59. 23511459

[123] Neemann K, Eichele DD, Smith PW, Bociek R, Akhtari M, Freifeld A. Fecal microbiota transplantation for fulminant Clostridium difficile infection in an allogeneic stem cell transplant patient. Transpl Infect Dis. 2012;14(6):E161–165. doi:10.1111/tid.12017. 23121625

[124] De Castro CG Jr., Ganc AJ, Ganc RL, Petrolli MS, Hamerschlack N. Fecal microbiota transplant after hematopoietic SCT: report of a successful case. Bone Marrow Transplant. 2015;50(1):145. doi:10.1038/bmt.2014.212. 25265462

[125] Mittal C, Miller N, Meighani A, Hart BR, John A, Ramesh M. Fecal microbiota transplant for recurrent Clostridium difficile infection after peripheral autologous stem cell transplant for diffuse large B-cell lymphoma. Bone Marrow Transplant. 2015;50(7):1010. doi:10.1038/bmt.2015.85. 25893454

[126] Webb BJ, Brunner A, Ford CD, Gazdik MA, Petersen FB, Hoda D. Fecal microbiota transplantation for recurrent Clostridium difficile infection in hematopoietic stem cell transplant recipients. Transpl Infect Dis. 2016;18(4):628–633. doi:10.1111/tid.12550. 27214585

[127] Moss EL, Falconer SB, Tkachenko E, Wang M, Systrom H, Mahabamunuge J, Relman DA, Hohmann EL, Bhatt AS. Long-term taxonomic and functional divergence from donor bacterial strains following fecal microbiota transplantation in immunocompromised patients. PLoS One. 2017;12(8):e0182585. doi:10.1371/journal.pone.0182585. 28827811 PMC5565110

[128] Kakihana K, Fujioka Y, Suda W, Najima Y, Kuwata G, Sasajima S, Mimura I, Morita H, Sugiyama D, Nishikawa H, Hattori M, et al.. Fecal microbiota transplantation for patients with steroid-resistant acute graft-versus-host disease of the gut. Blood. 2016;128(16):2083–2088. doi:10.1182/blood-2016-05-717652. 27461930 PMC5085256

[129] Qi X, Li X, Zhao Y, Wu X, Chen F, Ma X, Zhang F, Wu D. Treating steroid refractory intestinal acute graft-vs.-host disease with fecal microbiota transplantation: a pilot study. Front Immunol. 2018;9:2195. doi:10.3389/fimmu.2018.02195. 30319644 PMC6167440

[130] Kaito S, Toya T, Yoshifuji K, Kurosawa S, Inamoto K, Takeshita K, Suda W, Kakihana K, Honda K, Hattori M, Ohashi K. Fecal microbiota transplantation with frozen capsules for a patient with refractory acute gut graft-versus-host disease. Blood Adv. 2018;2(22):3097–3101. doi:10.1182/bloodadvances.2018024968. 30446486 PMC6258918

[131] van Lier YF, Davids M, Haverkate NJE, de Groot PF, Donker ML, Meijer E, Heubel-Moenen F, Nur E, Zeerleder SS, Nieuwdorp M, Blom B, Hazenberg MD. Donor fecal microbiota transplantation ameliorates intestinal graft-versus-host disease in allogeneic hematopoietic cell transplant recipients. Sci Transl Med. 2020;12(556). doi:10.1126/scitranslmed.aaz8926 32801142

